# Pre-harvest climate and post-harvest acclimation to cold prevent from superficial scald development in Granny Smith apples

**DOI:** 10.1038/s41598-020-63018-3

**Published:** 2020-04-10

**Authors:** Mathieu Marc, Maryline Cournol, Sylvain Hanteville, Anne-Sophie Poisson, Marie-Charlotte Guillou, Sandra Pelletier, François Laurens, Christine Tessier, Claude Coureau, Jean-Pierre Renou, Mickaël Delaire, Mathilde Orsel

**Affiliations:** 1 IRHS-UMR1345, Université d’Angers, INRAE, Institut Agro, SFR 4207 QuaSaV, 49071 Beaucouzé, France; 2 Station d’expérimentation fruitière La Morinière, 37800 Saint-Epain, France; 3 Ctifl Centre technique interprofessionnel des fruits et légumes, 37800 Saint-Epain, France

**Keywords:** Plant molecular biology, Abiotic

## Abstract

Superficial scald is one of the most serious postharvest physiological disorders that can affect apples after a prolonged cold storage period. This study investigated the impact of pre- and post-harvest climatic variations on superficial scald in a susceptible apple cultivar. Fruit batches with contrasting phenotypes for superficial scald incidence were identified among several years of “Granny Smith” fruit production. The “low scald” year pre-harvest climate was characterised by a warm period followed by a sudden decrease in temperature, playing the part of an *in vivo* acclimation to cold storage. This was associated with many abiotic stress responsive genes which were induced in fruit peel. In particular 48 Heat Shock Proteins (HSPs) and 5 Heat Shock transcription Factors (HSFs) were strongly induced at harvest when scald incidence was low. For “high scald” year, a post-harvest acclimation of 1 week was efficient in reducing scald incidence. Expression profiles of stress related genes were affected by the acclimation treatment and indicate fruit physiological adaptations to cold storage. The identified stress-responsive genes, and in particular HSPs, could be useful indicators of the fruit physiological status to predict the risk of scald occurrence as early as harvest.

## Introduction

Apple (*Malus domestica* Borkh.) is an economically important fruit crop, which is cultivated worldwide, with high nutritional value and a wide range of tastes. Apple fruit quality is affected by complex processes depending on genetic, environmental and agronomic factors, such as temperature, light, humidity or harvest stage^1^. To maximize their postharvest qualities, fruit are harvested early and stored at low temperature for several months. However, extended cold storage periods may lead to several physiological disorders such as superficial scald on ‘Granny Smith’, a sensitive variety. The appearance of brown patches on the epidermis and hypodermal cortical tissues during shelf life severely deteriorate fruit visual qualities, rendering the product unmarketable^2^.

The development of superficial scald symptoms is assumed to be an oxidative response due to cold stress during storage and the accumulation of oxidative products in apple peel^3^. Several studies have linked the acyclic sesquiterpene *α*-farnesene, a volatile compound accumulated in the peel under cold stress, and its auto-oxidation in conjugated trienols (Ctols) and ketone 6-methyl-5-hepten-2-one (MHO) with superficial scald symptoms^2,4^. Other studies have shown that superficial scald could also be induced even at low concentrations of *α*-farnesene^5^ and therefore suggested the involvement of other pathways. In particular, the oxidative stress from prolonged cold storage was considered to influence scald development^2^. New hypotheses arose from the study of the inhibitory effect of the ethylene inhibitor 1-MCP on scald development. It was shown that in addition to reduced ethylene and *α*-farnesene production, 1-MCP also reduced the accumulation of reactive oxygen species (ROS) in fruit peel, probably by promoting cell-membrane integrity^6^. Further studies revealed that 1-MCP stimulates the production of ROS scavengers, synthesis of fatty acids that could stabilize plastid and vacuole membranes against cold, and the accumulation of sorbitol that can act as a cryoprotectant^7^. In the proposed model, ROS production in response to cold stress plays a central role in scald development by disrupting membrane integrity and promoting the accumulation of phenolic compounds. The symptomatic peel browning would result from the enzymatic oxidation of the accumulated chlorogenic acid by the polyphenol oxidase (PPO), both initially in separate compartments^8^.

Although superficial scald was intensively studied for several years, its molecular or biochemical determinism was mainly analysed after long time cold storage during symptom development^3,9^, or under 1-MCP or DPA treatments^4,7,8,10–12^. Thus, early determinisms linked to the pre-harvest conditions, in particular to climate or fruit maturity stages remain poorly understood.

Maturity stage at harvest appears to be a key factor controlling scald development because immature apple fruit are more susceptible to develop symptoms. It has been speculated that the reason is a lower anti-oxidant to oxidant products ratio in immature apples^13^. There is also wide agreement that warm and dry growing periods contribute to increase susceptibility to superficial scald^2^. Therefore climate change could lead to an increase of scald incidence and severity through rising temperatures^14^. Although controversial and depending on several climatic factors, in certain locations the risk of superficial scald occurrence has been shown to be reduced when fruit were exposed to 150 hours below 10 °C during 4 or 6 weeks before harvest^15^. It was suggested that this ‘*in vivo*’ cold acclimation could modify the physiological status of the fruit at harvest, and thus preventing scald development during subsequent cold storage^16^. Based on the same idea, one experiment with step-wise cooling of harvest fruit has shown to reduce scald development for ‘Granny Smith’^17^.

Plant cold acclimation is associated with multiple physiological modifications participating in membrane stabilization and ROS reduction to maintain cellular homeostasis and prevent low temperature-induced oxidative injuries^18^. Studies have shown that pre-storage cold acclimation treatments can reduce chilling injury of harvested fruit for cucumber^19^, citrus^20^, zucchini^21^, avocado^22^, loquat^23^ and mango^24^. Production of protective compounds such as sorbitol could act as a cryoprotectant to promote directly membrane stabilization as demonstrated in transgenic *Arabidopsis*^7^. Gene expression and enzymatic activities of ROS scavengers such as superoxide dismutase (SOD), catalase (CAT) and ascorbate peroxidase (APX), as well as ascorbic acid and glutathione were also enhanced under cold acclimation as reported in cucumber^19^ and kiwi fruit^25^. Therefore, pre- or post-harvest cold acclimation could promote fruit resistance to oxidative stress triggered by cold storage and limit the development of scald symptoms.

The aim of this study was to investigate the effect of pre-harvest climate or post-harvest cold acclimation on superficial scald development in ‘Granny Smith’, and to identify its early molecular determinism. Fruit batches with contrasting phenotypes for superficial scald incidence and severity were identified among several years of fruit production. The “low” and “high” scald years were then analysed for their pre-harvest climatic variables, and transcriptomic analyses were set up between the corresponding fruit peel samples collected at harvest. The expression of identified genes and indicators of fruit physiological status were analysed in response to a post-harvest cold acclimation that was efficient in reducing scald incidence. The relevance of the identified genes is discussed with respect to their involvement in superficial scald determinism and prediction.

## Materials and methods

### Plant material and sampling strategies

**Figure 1:**
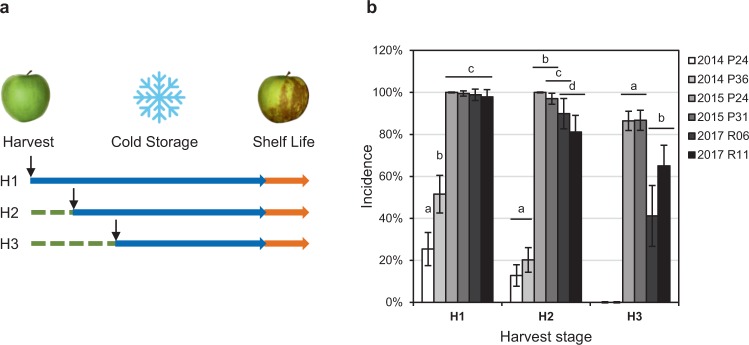
Effect of year and fruit maturity on superficial scald incidence. **(a)** Schematic representation of the experimental design. Fruit were harvested at three different maturity stages, early (H1), optimal (H2) or late (H3), stored under cold controlled atmosphere (blue arrow) for 5 to 6 months, and phenotyped for scald incidence after 1 week of shelf-life at room temperature (orange arrow). **(b)** Annual incidence of superficial scald injuries according to maturity at harvest on fruit collected from two different orchards each year. Values are binomial proportions and confidence intervals for n = 100 to 200 and α = 0.05. Snowflake image unchanged according to https://commons.wikimedia.org/wiki/File:Emojione_2744.svg, (https://creativecommons.org/licenses/by-sa/4.0/deed.en).

‘Granny Smith’ apple fruit (*Malus domestica* Borkh.) were harvested in 2014, 2015 and 2017 from commercially run orchards at the *Station Experimental de La Morinière* (30 ha estate, Saint Epain, France). Each year, fruit from two different orchards were harvested at three different maturity stage based on starch index (SI): early (H1, SI = 3), optimal (H2, SI = 5) and late (H3, SI = 7) (Fig. 1a; harvest dates in Supplementary Table S1). Fruit were collected from orchards P24 and P31 in 2014, P24 and P36 in 2015, R06 and R11 in 2017. For each batch, 100 to 200 fruit were randomly collected on both side of the orchard ranks, at human height, from at least 15 trees. Fruit were then stored at the *Station Experimental de La Morinière* in controlled atmosphere cold rooms (0.5 °C, 2% O_2_, 2% CO_2_ in 2014, 0.5 °C, 1.5% O_2_, 1% CO_2_ in 2015 and 2017) for 5 to 6 months. Each year peel samples were collected immediately at harvest stage.

An acclimation test was run on fruit collected in 2017 on R06 and R11 orchards. It consisted in 1 week storage at 8 °C (H1-acclim) before transfer to classic cold chambers at 2 °C up to 3, 4 and 5 months. For comparison, additional fruit batches harvested at H1 and H2 were immediately stored in classic cold chambers at 2 °C for 3, 4 and 5 months. Peel samples were collected after one day (1D) and one week (1 W) of acclimation or classic cold storage.

For each sample, peel was collected from both sun-exposed and shaded sides of 20 randomly selected fruit, immediately frozen, ground and homogenized in liquid nitrogen. Sample aliquots were stored at −80 °C before use.

### Evaluation of superficial scald symptoms

Following cold storage, fruit were phenotyped for the development of superficial scald symptoms immediately and after 1 week of shelf life at room temperature (20 °C). Incidence (percentage of fruit with symptoms) and severity were assessed for 100 to 200 fruit per batch. Severity was recorded for each fruit according to the relative surface area affected by symptoms using a 0 to 4 scale as following: S0, no symptom; S1, > 0% to 25%; S2, > 25% to 50%; S3, > 50% to 75%; S4, > 75% of affected surface area (Supplementary Fig. S1). Binomial proportion confidence intervals were calculated using the Wald method with continuity correction and error rate α = 0.05.

### Meteorological data analysis

**Figure 2:**
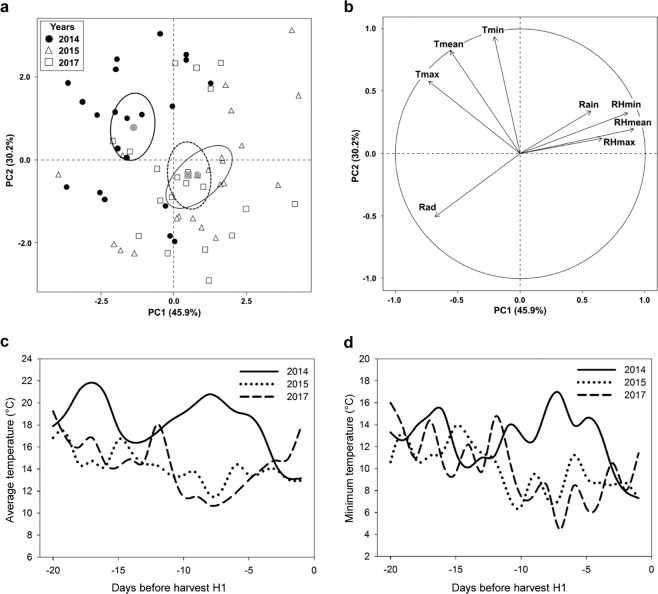
Analysis of pre-harvest climatic variables. PCA analysis of daily temperature (T, °C), rain (mm), relative humidity (RH) and radiation (Rad, W m^−2^) variables collected in the orchard each year for 20 d before harvest. Individuals (**a**) and variables (**b**) are represented in the first two dimension of the PCA. Pre-harvest daily temperatures: (**c**) average (Tmean, °C) and (**d**) minimum (Tmin, °C).

Meteorological data were collected at the *Station Experimental de La Morinière* (Saint Epain, France) using an Agriscope weather station (http://www.agriscope.fr/). Temperature, radiation, relative humidity and pluviometry were recorded hourly. Daily data synthetized as average, minimum and maximum were analysed over the 60 d preceding the fruit harvest. PCA analyses were performed for data collected 60 d, 40 d or 20 d before harvest using the R package FactoMineR^26^ (Fig. 2).

### RNA extraction, amplification and microarray hybridization

Total RNA was extracted from 3 × 10^−3^ kg FW of frozen fruit peel tissue finely ground in liquid nitrogen using two successive extraction procedures with CTAB and then SSTE buffers as described by Segonne *et al*.^27^. RNA quality indicator (RQI) was determined using the Experion TM RNA StdSens Analysis Kit (Bio-Rad, USA), only samples with RQI > 8 were kept for hybridizations and RT-qPCR experiments.

mRNAs were amplified from 200 ng of total RNA and labelled with either Cyanine-3 or Cyanine-5 fluorescent dye with the Agilent Low Input Quick Amp Labelling kit (Agilent, Foster City, CA, USA) according the manufacturer’s recommendations. Labelled samples were purified with Qiagen RNeasy kit (Qiagen, USA), combined as 20 pmol for each dye and co-hybridized to the Agilent microarray AryANE_v2 containing 60-mers oligonucleotide probes designed for each MDP gene from Velasco *et al*.^28^ *Malus domestica* genome v1, as described by Celton, Gaillard *et al*.^29^.

**Figure 3:**
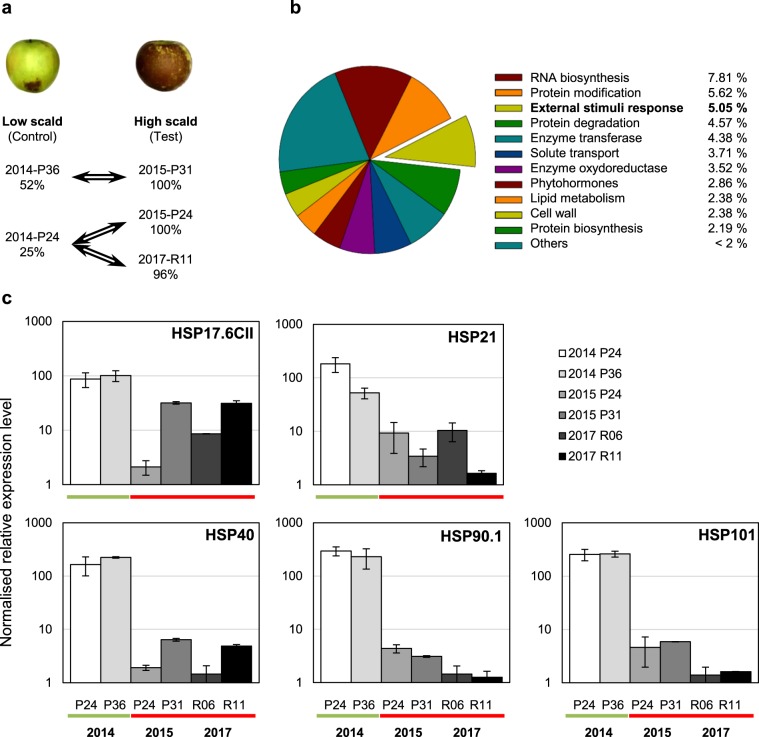
Transcriptomic analysis at harvest for fruit batches with contrasted phenotype for superficial scald. (**a**) Three comparisons were setup in dye-swap between peel samples collected at early harvest from fruit batches with “low” versus “high” scald incidence after cold storage and shelf-life. The three comparisons were combined in one statistical analysis setting the “low” scald samples as “control”, and “high” scald samples as “test”. (**b**) DETs assignment to functional categories: 1050 DETs with BH < 0.05 were selected and 57% were assigned (based on Mapman ontology using the Mercator web tool). (**c**) Relative gene expression level (RT-qPCR) in fruit peel samples collected at harvest from “low” (2014) or “high” (2015 and 2017) scald years for HSP17.6CII (*MD15G1053800*), HSP21 (*MD13G1108500*), HSP40 (*MD10G1289200*), HSP90.1 (*MD01G1208700*) and HSP101 (*MD06G1201600*). Data are mean values ± SD of n = 3.

The experimental design included three comparisons between samples collected at harvest from fruit batches with low *versus* high incidence for superficial scald (Fig. 3a). Each comparison included a technical repetition with dye swap. Thus, fruit peel samples were compared as following: samples 2014-P24 *vs* 2015-P24 (C1), samples 2014- P36 *vs* 2015-P31 (C2), samples 2014-P24 *vs* 2017-R11 (C3).

### Microarrays analysis

The Agilent Feature Extraction 11.5 software was used to extract data files from the scanned images obtained using the MS 200 microarray scanner (Roche Nimblegen). All statistical analyses were conducted based on a dye swap approach as described by Celton, Gaillard *et al*.^29^ with the R software^30^. Briefly, for each comparison (C1 to C3) data were normalized with the lowess method, and differential expression analyses were carried out using the lmFit function and the Bayes moderated t test using the R package LIMMA^31^ from the Bioconductor project. To estimate gene expression levels, the normalized expression values were corrected from background. An additional statistical analysis, including a Benjamini–Hochberg procedure in order to controls the false discovery rate (FDR), was performed with combining the three initial comparisons (C1 to C3) considered as three biological repetitions.

Probes were considered reporting differentially expressed transcripts when their respective corrected P-value (BH) was equal or below 0.05. Only probes reporting sense transcription (96.3%) were then considered in this study. In order to take into account the improvements brought by the GDDH13 v1.1 assembly and annotation^32^, specific and 100% matching targets were search with blast for the selected AryANE_v1 probes. Best blast search results were reported in the Supplementary File S1 under the “spe_new” column. The “spe” code correspond to the identification of at least one target with 100% match among the GDDH13 v1.1 transcripts (76% of the selection). Only the identified set of MD GDDH13 v1.1 genes was then considered in the subsequent analyses and RT-qPCR experiments. Functional classification of DETs was based on Mapman ontology using the Mercator web tool (http://mapman.gabipd.org/web/guest/app/mercator)^33^. An enrichment analysis (Wilconxon rank sum test with Benjamin-Hochberg-correction) was performed with Mapman software 3.6.0^34^. Arabidopsis genes GO annotations were retrieved from The Arabidopsis Information Resource (TAIR) on www.arabidopsis.org. The microarrays data are available under the accession number GSE135863 in the Gene Expression Omnibus database (https://www.ncbi.nlm.nih.gov/geo/).

### Reverse transcription and quantitative real time PCR (RT-qPCR)

Total RNA samples used for the microarrays experiments and RNA extracted from the 2017 acclimation experiment peel samples were treated with 1 U of DNAse I (Promega, USA) and cDNAs were synthesized from 1 μg of DNA-free-RNA with oligo(dT) 15 and 200 U of MMLV-RT (Promega) according to Segonne *et al*.^27^. For each cDNA, qPCR experiments were carried out in triplicate according to Segonne *et al*.^27^ using the IQ SYBR Green Super Mix (Biorad) and specific forward and reverse primers at 0.3 µM. Amplifications were performed on a CFX Maestro^TM^ Real-Time PCR instrument (Bio-Rad, USA) and data analysed with CFX Maestro™ Software (Bio-Rad, version 1.1).

Primer pairs were designed for short and specific amplification based on GDDH13 v1.1 genome sequence (Supplementary Table S2) using Primer3Plus (http://www.bioinformatics.nl/cgi-bin/primer3plus/primer3plus.cgi) or retrieved from Vergne *et al*.^35^. Each primer pair was tested for its respective specificity and efficiency of the reactions: (1) amplicons were sequenced once and aligned to GDDH13 v1.1; (2) for each run, single product amplification was confirmed by melting curve analyses. Amplification efficiency was assessed using a dilution curve method over a 6 point dilution series. Only primer pairs with efficiencies higher than 85% were retained for further analysis.

Based on microarray results, three reference genes never differentially expressed in all comparisons were selected as reference genes to calculate a normalization factor (geometric mean). Relative expression levels were calculated using a formula derived from the 2–ΔΔCt method [ΔCt = (Ctgoi–Ctref)], where Ct is the threshold cycle, goi is the gene of interest, and ref is the reference gene^36^. Standard deviations were calculated from three replicates following the error-propagation rule formula.

## Results

### Superficial scald symptoms development

Superficial scald was evaluated for different maturity stages at harvest (H1 to H3) on 2 orchards from the same location over 3 years (Fig. 1a). Each year scald incidence decreased while fruit maturity at harvest increased. In particular in 2014 no symptom was detected on late H3 fruit, while 25% and 52% of the early H1 fruit, and 12% and 13% of the optimum H2 fruit were affected by scald (Fig. 1b). Scald incidence was significantly higher in 2015 and 2017 (Fig. 1b), with scald incidence reaching 98% to 100% at H1 and remaining above 80% at H2. 2015 and 2017 fruit were also more severely affected than in 2014 (Supplementary Fig. S1). In 2015 incidence only slightly decreased to 90% at the latest harvest stage H3 while, as observed in 2014, 2017 incidence and severity progressively decreased with higher maturity stage at harvest (Fig. 1b and Supplementary Fig. S1). However, 2017 incidence remained higher than in 2014. Therefore, 2014 was a “low scald” year, while 2015 and 2017 were “high scald” years.

### Analysis of pre-harvest climate

Climatic variables were retrieved from the weather station located in the location of the orchards in order to analyse pre-harvest conditions each year. The total number of hours when hourly temperature was below 10 °C (NH10) was calculated each year from 60 d, 45 d, 30 d and 20 d before harvest (Table 1). Irrespective of the pre-harvest period considered, the NH10 was lower than 150 hours in all years. Contrary to expectation^15^, NH10 was the lowest for the “low scald” year 2014. Based on these results, principal component analysis (PCA) was performed for each previously cited period to identify climatic differences between years. The analysis performed for the period of 20 d before harvest showed the maximum difference between the years (Fig. 2a). The first two components of the PCA captured most of the variability accounting for respectively 46% and 30% of explained variance (Fig. 2b). These two components allow the clear separation of pre-harvest days between “low scald” year 2014 and “high scald” years 2015 and 2017. The 2014 pre-harvest days were negatively correlated with PC1 and positively correlated with PC2. PC1 was mainly defined by relative humidity (RH), in particular by the daily mean and minimum RH (RH_mean_ correlation 0.91, P-value = 5 × 10^−25^; RH_min_, correlation 0.86, P-value = 1.4 × 10^−19^). PC2 included variables linked to the daily temperature, in particular the minimum and the mean temperature of the day (T_min_, correlation 0.94, P-value 2.6 × 10^−29^; T_mean_, correlation 0.82, P-value 1.5 × 10^−16^). Pre-harvest days were thus dryer and warmer in the “low scald” year of 2014 than in the “high scald” years of 2015 and 2017. Analysis of the daily average temperature confirms that the pre-harvest period was overall warmer in 2014 than in 2015 and 2017 (Fig. 2c). However three days before harvest the mean temperature dropped suddenly from 18 °C to 13 °C. This rapid decrease was especially marked for the daily minimum temperature that dropped from 15 °C to 7 °C (Fig. 2d). A drastic shift of temperature also occurred just before 2017 harvest, a “high-scald” year, but in the opposite way with a rapid increase of the mean and minimum daily temperatures. The low scald incidence observed only in 2014 could be explained by an “*in vivo*” acclimation to cold due to the pre-harvest warm period followed by a rapid decrease in temperature.

**Table 1:** Number of pre-harvest hours with mean temperature below 10 °C (NH10).

Numbers of days	NH10		
	2014	2015	2017
60	40	99	96
45	40	99	96
30	18	99	95
20	18	72	82

### Transcriptome analysis at harvest

In order to identify early determinants of scald development, transcriptomic analyses were performed at early harvest stage on the base of scald contrasted phenotypic data after cold storage. Expression profiles of apple peel sampled at harvest from low scald year 2014 were compared with the high scald years 2015 and 2017. Expression profiles of 2014 peel samples from P24 and P36 orchards were compared respectively with expression profiles of 2015 peel samples from P24 and P31 orchards. An additional comparison was set up between 2014-P24 and 2017-R11 peel samples (Fig. 3a). We hypothesized that genes involved in early events that prevent or induce scald development displayed similar differential expression patterns for each comparison. Therefore, we set up an additional t-test combining the three comparisons. 1491 differentially expressed transcripts (DETs) were identified with significant BH adjusted P-value (BH < 0.05). Thus, 1435 sense DETs were selected, of which 41.7% were up regulated and 58.3% were down regulated in high scald samples, when compared to low scald samples (Supplementary File S1).

A search for corresponding genes on the *Malus domestica* GDDH13 v1.1 genome version^32^ led to the identification of 1050 (73.2%) non-redundant genes. Based on MD gene annotation, 57.1% of these DETs were assigned to functional categories (Fig. 3b; Supplementary File S3). Among them, the most represented were “RNA biosynthesis” (7.81%), “Protein modification” (5.62%), “External stimuli response” (5.05%) and “Protein degradation” (4.57%). It is noteworthy that 42.9% of the DETs were not allocated to any category, and that 7.90% belonged to enzyme categories “oxidoreductase” or “transferase”. In order to validate data obtained from the microarray analysis, the relative transcript abundances of a subset of differentially expressed genes with contrasted expression profiles were tested by RT-qPCR using cDNA from low or high scald samples (Supplementary File S4). The results were consistent with those obtained from the microarray analysis (Pearson correlation coefficient = 0.83).

### Candidate genes associated with later high scald symptoms

The most significantly over-represented category was “External stimuli response” (P-value = 8.61 × 10^−14^) and more specifically “response to temperature” (3.81% of DETs, P-value = 3.93 × 10^−17^) (Supplementary File S3). It included members from all known Heat Shock Protein families (HSP100, HSP90, HSP70, HSP60 and sHSP) (Table 2). All 48 DETs belonging to these families were down regulated in 2015 and 2017 when scald incidences were high. The 10 most differentially expressed transcripts of the analysis are potential HSPs, in addition 64% of the top 50 DETs are HSPs (Supplementary File S1). In particular, we verified that potential orthologous genes to Arabidopsis HSPs known to be induced in response to heat stress were down regulated in “high scald” samples: *MD15G1053800* and *MD13G1108500* respectively coding for small HSPs HSP17.6CII and HSP21, as well as *MD01G1208700* and *MD06G1201200* coding for HSP90.1 and HSP101^37–40^ (Fig. 2c). Furthermore, 74% of the DETs annotated as HSPs could be linked to a gene ontology (GO) annotation related to heat stress (Supplementary File S1). Most of them were also annotated as responding to light stimulus and/or oxidative stress. The subgroup of Heat shock transcription factors (HSFs) was also significantly over represented in the transcriptomic analysis. A potential *HsfA2*, activator of transcription for many HSPs in Arabidopsis^41^, and its potential targets were particularly repressed in “high scald” samples (Table 2). This transcription factor has been shown to respond to different environmental stresses, including the combination of heat and high light, as well as hydrogen peroxide (Supplementary File S1).

**Table 2:** HSP and HSF transcripts down regulated in “high scald” fruit peel.

Seq_id	LR	MD gene	TAIR				
			evalue	name	Annotation	AtHSP	GO
***Small HSP (sHSP)***							
MDP0000700383	−3.26	MD15G1053800	3 × 10^−59^	AT5G12020	17.6 kDa class II heat shock protein	AtHsp17.6-CII	HLOx
MDP0000362505	−2.43	MD08G1068200	3 × 10^−58^	AT5G12020	17.6 kDa class II heat shock protein	AtHsp17.6-CII	HLOx
MDP0000188935	−3.00	MD08G1068000	9 × 10^−57^	AT5G12020	17.6 kDa class II heat shock protein	AtHsp17.6-CII	HLOx
MDP0000214382	−3.79	MD13G1108500	3 × 10^−37^	AT4G27670	21 kDa class II heat shock protein	AtHsp25.4-P*	HLOx
MDP0000125300	−2.68	MD06G1060300	3 × 10^−54^	AT4G25200	23.6 kDa class II heat shock protein	AtHsp23.6-M*	
MDP0000795157	−2.62	MD10G1289200	0	AT2G20560	DNAJ heat shock family protein		
MDP0000290546	−2.58	MD05G1310300	6 × 10^−179^	AT2G20560	DNAJ heat shock family protein		
MDP0000549793	−1.95	MD12G1172300	0	AT3G08970	DNAJ protein ERDJ3A		H
MDP0000164489	−2.70	MD11G1089300	1 × 10^−69^	AT1G07400	HSP20-like		HOx
MDP0000493154	−2.94	MD11G1087100	3 × 10^−69^	AT1G07400	HSP20-like		HOx
MDP0000574524	−4.56	MD05G1240300	3 × 10^−38^	AT1G07400	HSP20-like		HOx
MDP0000094857	−3.22	MD11G1089400	3 × 10^−16^	AT1G07400	HSP20-like		HOx
MDP0000791550	−1.74	MD07G1210400	2 × 10^−78^	AT1G53540	HSP20-like	AtHsp17.6C-Cl*	HOx
MDP0000265157	−2.59	MD01G1144400	5 × 10^−73^	AT1G53540	HSP20-like	AtHsp17.6C-Cl*	HOx
MDP0000412799	−2.08	MD07G1210800	2 × 10^−66^	AT1G53540	HSP20-like	AtHsp17.6C-Cl*	HOx
MDP0000424976	−1.64	MD11G1087200	2 × 10^−22^	AT1G53540	HSP20-like	AtHsp17.6C-Cl*	HOx
MDP0000152564	−2.45	MD17G1151000	4 × 10^−53^	AT1G54050	HSP20-like	AtHsp17.4-CIII*	HLOx
MDP0000136609	−3.02	MD15G1443700	2 × 10^−49^	AT4G10250	HSP20-like	AtHsp22.0-ER*	HOx
MDP0000656080	−2.97	MD08G1249100	2 × 10^−45^	AT4G10250	HSP20-like	AtHsp22.0-ER*	HOx
MDP0000208958	−1.46	MD09G1271100	2 × 10^−58^	AT5G37670	HSP20-like	AtHsp15.7-Cl*	HOx
***HSP60 - HSP70***							
MDP0000752314	−1.50	MD10G1170700	0	AT3G23990	Heat shock protein 60		H
MDP0000859313	−1.18	MD05G1182500	0	AT3G23990	Heat shock protein 60		H
MDP0000867730	−2.03	MD07G1196600	3 × 10^−39^	AT1G56410	Heat shock protein 70		H
MDP0000620433	−2.30	MD07G1197200	0	AT3G12580	Heat shock protein 70	AtHsp70-4*	HLOx
MDP0000122734	−2.27	MD17G1226000	0	AT3G12580	Heat shock protein 70	AtHsp70-4*	HLOx
MDP0000220559	−1.63	MD01G1126500	0	AT3G12580	Heat shock protein 70	AtHsp70-4*	HLOx
MDP0000311339	−3.00	MD15G1150500	0	AT1G16030	Heat shock protein 70B	AtHsp70-5*	H
MDP0000172536	−2.16	MD16G1192600	0	AT2G32120	Heat-shock protein 70T-2	AtHsp70-8*	HLOx
MDP0000684170	−1.83	MD13G1191900	0	AT2G32120	Heat-shock protein 70T-2	AtHsp70-8*	HLOx
***HSP90***							
MDP0000181929	−1.06	MD11G1037400	0	AT5G56000	Heat shock protein 81.4	AtHsp90-4	H
MDP0000254260	−3.53	MD01G1208700	0	AT5G52640	Heat shock protein 90.1	AtHsp90-1	H
MDP0000303430	−3.00	MD07G1279200	0	AT5G52640	Heat shock protein 90.1	AtHsp90-1	H
MDP0000948331	−2.38	MD00G1081900	6 × 10^−88^	AT5G52640	Heat shock protein 90.1	AtHsp90-1	H
***HSP 100***							
MDP0000217508	−3.59	MD06G1201600	0	AT1G74310	Heat shock protein 101	AtHsp100-1*	HLOx
MDP0000303015	−2.76	MD14G1211000	0	AT1G74310	Heat shock protein 101	AtHsp100-1*	HLOx
MDP0000308722	−2.32	MD06G1201200	0	AT1G74310	Heat shock protein 101	AtHsp100-1*	HLOx
MDP0000755970	−2.27	MD14G1210600	0	AT1G74310	Heat shock protein 101	AtHsp100-1*	HLOx
***Other related chaperone***							
MDP0000197501	−2.99	MD16G1124100	1 × 10^−21^	AT1G12060	BAG chaperone regulator		
MDP0000215062	−1.79	MD14G1054700	0	AT3G12050	Hsp90 binding protein		
MDP0000932255	−1.64	MD12G1055300	0	AT3G12050	Hsp90 binding protein		
MDP0000190008	−1.17	MD13G1024100	2 × 10^−53^	AT1G23100	Hsp10 Hsp60-co-chaperone		
MDP0000422652	−1.15	MD04G1081900	0	AT4G12400	Hsp70-Hsp90 organizing protein		HLOx
MDP0000161691	−1.08	MD06G1065400	0	AT4G12400	Hsp70-Hsp90 organizing protein		HLOx
*HSF*							
MDP0000527802	−1.50	MD02G1171800	2 × 10^−92^	AT4G36990	Heat shock factor 4	HsfB1	H
MDP0000925901	−1.64	MD03G1258300	2 × 10^−124^	AT3G22830	Heat shock transcription factor A6B	HsfA6b	H
MDP0000155667	−1.05	MD01G1198700	6 × 10^−79^	AT5G62020	Heat shock transcription factor B2A	HsfB2a	H
MDP0000243895	−2.80	MD15G1057700	8 × 10^−126^	AT2G26150	Heat shock transcription factor A2	HsfA2*	HLOx
MDP0000489886	−1.91	MD08G1064100	5 × 10^−120^	AT2G26150	Heat shock transcription factor A2	HsfA2*	HLOx

Reference sequence for probes design (Seq_id), Log ratio (LR) of differential analysis, corresponding GDDH13 gene (MD gene), Arabidopsis gene annotation (TAIR e-value, gene name and annotation), Arabidopsis short name according to Swindell *et al*.^77^ (AtHSP), Arabidopsis GO annotation (response do heat (H), light (L) or ROS (Ox)). *Gene regulated by AtHSFA2 according to Nishizawa *et al*.^41^.

Other transcription factors involved in the light stress response were similarly down regulated in samples from “high scald” fruit batches (Table 3), like the potential orthologous genes of the Arabidopsis bZIP genes *HY5* and *HYH* (*MD08G1147100* and *MD16G1132200*)^42^. *MD03G1297100* and *MD11G1316800*, two potential MYB12 regulators of the flavonoid pathways known to respond to high light^43^, were also down regulated. In agreement, a potential orthologous downstream regulated gene was also down regulated, namely a phenylalanine ammonia-lyase (PAL1, *MD12G1116700*). *MD03G1099300*, a potential *ZAT12* gene C2H2 transcription factor also named *RHL41* “responsive to high light 41”, was also down regulated. The Arabidopsis *ZAT12* gene (*AT5G59820*) was shown to be also induced by heat and cold stress and to be involved in cold and photosynthetic acclimation^44,45^ (Table 3; Supplementary File S1).

**Table 3:** Selected DETs associated with abiotic stress response.

Seq_id	LR	MD gene	TAIR				
			evalue	name	Short name	Annotation	GO
***Response to heat***							
MDP0000175388	−2.36	MD04G1022400	0	AT5G48570	ROF2	FKBP-type peptidyl-prolyl cis-trans isomerase	H
MDP0000275263	−2.00	MD09G1263000	5 × 10^−112^	AT1G66080		Protein of unknown function DUF775	H
MDP0000273764	−1.06	MD17G1258000	9 × 10^−116^	AT1G66080		Protein of unknown function DUF775	H
MDP0000917573	−1.68	MD02G1050200	5 × 10^−70^	AT3G24500	ATMBF1C	Multiprotein bridging factor 1 C	H
MDP0000129942	−1.39	MD15G1187400	2 × 10^−69^	AT3G24500	ATMBF1C	Multiprotein bridging factor 1C	H
MDP0000205111	−1.40	MD10G1156300	0	AT3G25230	ROF1	Rotamase FKBP1 protein folding catalyst	H
MDP0000141863	−1.04	MD05G1166200	0	AT3G25230	ROF1	Rotamase FKBP1 protein folding catalyst	H
***Response to light***							
MDP0000586302	−2.06	MD08G1147100	1 × 10^−66^	AT5G11260	HY5	bZIP transcription factor family protein	L
MDP0000388769	−1.96	MD12G1116700	0	AT2G37040	PAL1	PHE ammonia lyase 1	LOx
MDP0000268980	−1.68	MD11G1316800	3 × 10^−73^	AT2G47460	MYB12	Myb domain protein 12	L
MDP0000119725	−1.67	MD03G1297100	8 × 10^−73^	AT2G47460	MYB12	Myb domain protein 12	L
MDP0000281626	−1.41	MD10G1316100	0	AT1G61800	GPT2	Glucose-6-phosphate/phosphate translocator 2	L
MDP0000771031	−1.31	MD05G1305700	1 × 10^−135^	AT2G29120	ATGLR2.7	Glutamate receptor 2.7	L
MDP0000831937	−1.21	MD07G1285600	0	AT5G24120	SIGE	RNA polymerase sigma subunit E	L
MDP0000149332	−1.17	MD04G1144100	3 × 10^−90^	AT2G37970	ATHBP2	SOUL heme-binding family protein	L
MDP0000159766	−1.08	MD17G1265700	0	AT2G42690		alpha/beta-Hydrolases superfamily protein	L
***Response to cold***							
MDP0000299872	−1.15	MD09G1043700	0	AT5G40010	AATP1	AAA-ATPase 1	C
MDP0000317816	−1.13	MD04G1164500	8 × 10^−116^	AT5G01600	ATFER1	Ferretin 1	COx
MDP0000203813	−1.11	MD07G1204100	0	AT3G55580	RCC1	Regulator of chromosome condensation	C
***Response to multiple stimuli***							
MDP0000739699	−2.65	MD14G1150400	3 × 10^−50^	AT3G22840	ELIP	Chlorophyll A-B binding family protein	HLC
MDP0000446914	−1.87	MD17G1280400	0	AT2G47180	ATGOLS1*	Galactinol synthase 1	HLCOx
MDP0000595671	−1.18	MD03G1099300	3 × 10^−37^	AT5G59820	RHL41	ZAT12 C2H2-type zinc finger family protein	HLCOx
MDP0000226817	−1.27	MD10G1110200	0	AT1G17870	EGY3*	Zinc metallopeptidase	HLOx
MDP0000238942	−1.01	MD01G1185500	0	AT2G36530	LOS2	Enolase	LC
MDP0000755567	−1.17	MD03G1289900	1 × 10^−21^	AT4G02380	SAG21	Senescence-associated gene 21	LCOx
MDP0000286604	−0.89	MD17G1211100	4 × 10^−15^	AT5G20230	ATBCB	Blue-copper-binding protein	LCOx
MDP0000265874	−0.87	MD15G1253900	1 × 10^−11^	AT1G20440	COR47	Cold-regulated 47	HC
***Response to oxidative stress***/***ROS processing***							
MDP0000166359	−1.67	MD06G1110300	2 × 10^−98^	AT1G64500		Glutaredoxin family protein	Ox
MDP0000171695	−1.87	MD14G1014200	6 × 10^−42^	AT3G10020		Protein of unknown function	Ox
MDP0000566567	−2.42	MD05G1211000	8 × 10^−108^	AT1G78380	ATGSTU19	Glutathione S-transferase TAU 19	Ox
MDP0000187493	−1.76	MD05G1211100	9 × 10^−31^	AT1G78380	ATGSTU19	Glutathione S-transferase TAU 19	Ox
MDP0000459010	−1.46	MD05G1210700	8 × 10^−110^	AT1G78380	ATGSTU19	Glutathione S-transferase TAU 19	Ox
MDP0000755113	−1.03	MD05G1210000	5 × 10^−116^	AT1G78380	ATGSTU19	Glutathione S-transferase TAU 19	Ox
MDP0000135807	−1.15	MD05G1309500	4 × 10^−40^	AT1G28480		Thioredoxin superfamily protein	Ox
MDP0000179654	−0.96	MD10G1288100	2 × 10^−41^	AT1G28480		Thioredoxin superfamily protein	Ox
MDP0000340109	−1.26	MD15G1395600	1 × 10^−71^	AT1G22840	CYTC-1	CYTOCHROME C-1	Ox
MDP0000205322	−1.02	MD08G1213100	7 × 10^−72^	AT1G22840	CYTC-1	CYTOCHROME C-1	Ox
MDP0000248822	−1.15	MD15G1085500	0	AT2G26560	PLP2	Phospholipase A 2 A	Ox
MDP0000770103	−1.17	MD11G1004100	6 × 10^−137^	AT5G05340	PRX52	Peroxidase superfamily protein	Ox
MDP0000642594	−1.13	MD04G1139400	3 × 10^−80^	AT3G09270	ATGSTU8	Glutathione S-transferase TAU 8	Ox
MDP0000320982	−1.12	MD04G1139500	4 × 10^−83^	AT3G09270	ATGSTU8	Glutathione S-transferase TAU 8	Ox
MDP0000126107	−0.87	MD04G1104900	1 × 10^−154^	AT3G09640	APX2*	Ascorbate peroxidase 2	Ox
MDP0000326493	1.06	MD02G1013800	9 × 10^−33^	AT4G30380		Barwin-related endoglucanase	Ox
MDP0000126601	1.12	MD17G1039200	4 × 10^−27^	AT5G14920	GASA14	Gibberellin-regulated family protein	Ox

Reference sequence for probes design (Seq_id), Log ratio (LR) of differential analysis, corresponding GDDH13 gene (MD gene), Arabidopsis gene annotation (TAIR e-value, gene name and annotation), Arabidopsis GO annotation (response do heat (H), light (L), cold (C) or ROS (Ox)). *Gene regulated by *AtHSFA2* according to Nishizawa *et al*.^41^.

A few DETs responding to cold and/or involved in cold acclimation were identified (Table 3, Supplementary File S1). The most differentially expressed were down regulated in “high scald” samples, and as with *ZAT12* they were also annotated as responding to heat and light stimuli. In particular, *MD14G1150400*, a potential orthologue for *AT3G22840* coding for a chlorophyll binding protein ELIP1 involved in photoprotection was down regulated in “high scald” samples. The *MD17G1280400* gene, potentially coding for an orthologue of the AtGOLS1 a galactinol synthase induced by heat, light and cold stimulus, was also down regulated.

Finally, among the DETs with GO for response to oxidative stress or probably involved in redox cellular homeostasis, several genes potentially coding for glutathione S-transferase were down regulated (*MD05G1210700, MD05G1210000, MD05G1211000, MD05G1211100, MD04G1139400, MD04G1139500*) as well as potential ascorbate peroxidase APX2 (*MD04G1104900*), glutaredoxin (*MD06G1110300*) and thioredoxin (*MD05G1309500, MD10G1288100*) (Table 3; Supplementary File S1).

Interestingly, only 1.9% of the genes were related to the biotic stress response while 12.5% of the DETs had GO related to the abiotic stress response. The whole transcriptome profile suggested that “low scald” fruit have been exposed to abiotic stresses before harvest, probably a combination of thermal and high light stresses.

### Effect of post-harvest cold acclimation on scald

**Figure 4:**
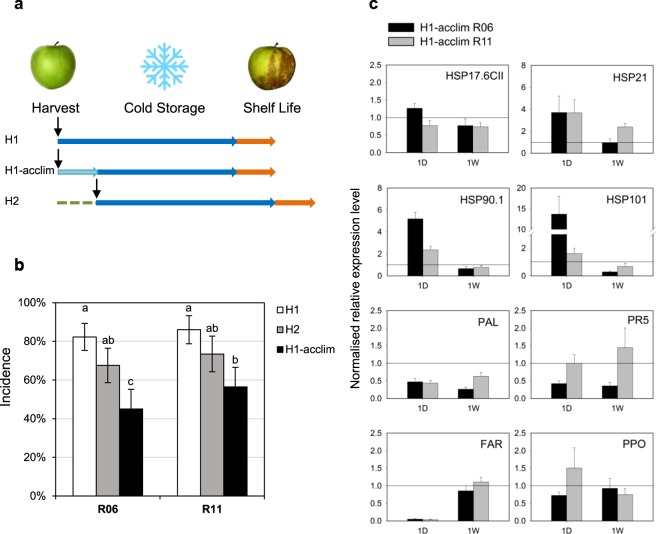
Effect of cold acclimation on superficial scald incidence. **(a)** Schematic representation of the experimental design. Early harvested fruit were cold acclimated at 8 °C for 1 week (H1-acclim, light blue) and compared to fruit not acclimated (H1) or harvested 1 week later (H2). Fruit were stored in classic cold conditions (blue arrow) and phenotyped for scald incidence after one week of shelf-life at room temperature (orange arrow). **(b)** Incidence of superficial scald injuries after 4 months cold storage according to acclimation treatment and harvest stage on fruit collected from two different orchards (R06 and R11). Values are binomial proportions and confidence intervals for n = 100 and α = 0.05. (**c**) Effect of acclimation on relative gene expression in fruit peel samples after one day (1D) and one week (1W). Expression relatively to classic cold storage for HSP17.6CII (*MD15G1053800*), HSP21 (*MD13G1108500*), HSP90.1 (*MD01G1208700*), HSP101 (*MD06G1201600*), PAL (*MD12G1116700*), PR5 (MD04G1064200), FAR (*MD10G1311000*) and PPO (*MD10G1299400*). Data are mean values ± SD of n = 3 for fruit collected in both orchards R06 and R11. Snowflake image unchanged according to https://commons.wikimedia.org/wiki/File:Emojione_2744.svg, (https://creativecommons.org/licenses/by-sa/4.0/deed.en).

H1 fruit harvested in 2017 were submitted to a post-harvest cold period of 1 week at low temperature prior to classic cold storage (Fig. 4a). When fruit were cold acclimated (H1-acclim), scald incidence was significantly reduced for both fruit batches dropping from 82% to 45% for R06, and from 86% to 56% for R11 after 4 months cold storage (Fig. 4b). Scald symptoms were also less severe for H1-acclim fruit than for H1 fruit (Supplementary Fig. S3). Scald incidence for H2 fruit, harvested one week later and immediately stored in classic cold was also reduced when compared to H1 fruit, but less than for the H1 acclimated fruits. This intermediate phenotype for H2 fruit was not observed any more after 5 months. (Supplementary Fig. S3). Therefore, the observed reduced scald incidence for H1-acclim fruit was not only due to more mature fruit (1 week delay before classic cold storage), but principally due to the cold acclimation treatment.

### Effect of post-harvest cold acclimation on physiological stress indicators

As many HSPs responding to various stresses were found induced in 2014, the effect of cold acclimation on their expression was analysed. When compared with classic cold storage (H1 samples), the identified MD genes coding for HSP21, HSP90 and HSP101 were up-regulated after 1 day but not after 1 week of acclimation (Fig. 4c). Cold acclimation conditions tended to limit the major down regulation of HSP genes immediately observed after 1 day when fruit were stored in classic cold conditions (Supplementary Fig. S4). After one week of acclimation, all tested HSPs gene expression were similarly repressed as in classic cold conditions.

The effect of cold acclimation was further analysed using biotic and abiotic stress responsive gene expression^46^. The cold acclimation treatment had a repressive effect on *MD12G1116700* gene coding for a phenyl ammonia lyase (PAL) (Fig. 4c). PAL was immediately induced by classic cold storage for 1 week, while the cold acclimation treatment limited its induction for the entire first week of storage (Supplementary Fig. S4). In contrast, for *MD04G1064200*, a PR5 Thaumatin coding gene, the acclimation had a clear repressive effect only for the R06 fruit batch (Fig. 4c). PR5 was progressively induced under classic cold storage, indicating an increasing fruit stress status (Supplementary Fig. S4).

The qPFD chip also included FAR *(MD10G1311000)*, coding for a terpene synthase probably involved in the pathway leading to the biosynthesis of *α*-farnesene, and PPO *(MD10G1299400)*, coding for a polyphenol oxidase. Both genes are reporters of enzymatic activities suspected of being involved in the appearance of scald symptoms. FAR and PPO were progressively induced by cold storage (Supplementary Fig. S4). The acclimation treatment transiently inhibited the strong induction of FAR on the first day but did not have a consistent effect on PPO (Fig. 4c) which was also induced in both conditions (Supplementary Fig. S4).

## Discussion

Our study clearly demonstrated that pre-harvest ‘*in vivo*’ acclimation to cold is not necessarily due to the accumulation of cold temperature for 2 months before harvest as generally accepted, but also by a thermal variation due to a warm period followed by sudden cold temperatures three weeks before harvest. This thermal acclimation induced an increase in expression of HSP genes as well as other genes involved in abiotic stress responses. This transcriptomic response probably induced a physiological adjustment allowing the fruit to withstand cold storage and limiting further scald development.

The putative HSPs and HSFs apple orthologues highly expressed in low scald samples were probably induced by the high temperatures observed specifically through 2014 pre-harvest period. Indeed, the induction of HSP gene expression and proteins has already been observed in fruit flesh exposed to high daily temperatures in orchards^47^, and some HSF genes were shown to respond to heat stress in different apple tissues^48^. The sudden change of weather for cold temperatures three days before harvest may have also contributed to the induction of some HSPs. In particular, members of the HSP70 family were shown to be specifically induced by cold stress in spinach leaves and Arabidopsis^49,50^. Cold stress generates oxidative stress responses, and many AtHSPs were shown to be induced by ROS (Table 2). Therefore MdHSP expression could also have been indirectly induced by cold *via* ROS production.

**Figure 5:**
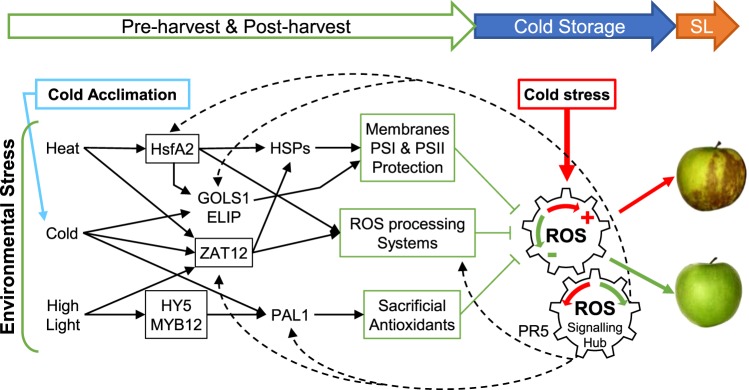
Hypothetical model for pre- and post-harvest climatic effects on superficial scald incidence. Pre-harvest environmental stress and post-harvest cold acclimation activate interconnected regulation pathways (black arrows, transcription factors in black boxes). Fruit physiological status is therefore modified, in particular its redox status (physiological process in green boxes), which can counteract the oxidative stress due to cold storage (red arrow) and limit scald. The model involves the integration of the different signals at the ROS production level and feed-back through the ROS signalling hub^76^ (dash arrow). Sacrificial antioxidants include housekeeping compounds such as proline, as well as secondary compounds such as flavonoids^56^. The ROS processing system involves compounds and enzymes allowing repeated redox-cycling such as those involved in the glutathione-ascorbate cycle^71^. PR5 gene expression may be correlated with the increasing stress status of fruit during cold storage. PSI and PSII, photosystems I and II; ROS reactive oxygen species; SL, shelf life.

The major negative effect of cold stress is that it induces severe membrane damage, alters the photosynthetic electron transfer machinery, and generates oxidative stress^18^. HSPs probably protect the cells in regulating the plant antioxidant system and ROS production^51^. Several chloroplastic sHSPs were shown to protect the Photosystem II (PSII) against formation of oxygen and photoinhibition in various stress conditions^52,53^. In particular, the tomato HSP21 and the sweet pepper CaHSP26 protect PSII from temperature dependent oxidative stress^39,54^. It was recently shown that the spinach SoHSC70 positively regulates thermotolerance by alleviating cell membrane damage, reducing ROS accumulation, and improving activities of antioxidant enzymes^55^. We can then hypothesize that among the identified apple HSFs and HSPs induced by pre-harvest conditions in 2014, several of them could have protected the fruit against cold stress induced by storage by reducing the oxidative stress and stabilizing the cell membranes (Fig. 5).

The protective effect of HSPs was also probably enhanced by the regulation network depending on HsfA2. Indeed, AtHsfA2 was identified as a key regulator in response to several types of environmental stress, inducing many HSPs but also genes involved in various cellular adaptation processes such as ascorbate peroxidase 2 (*APX2*) and galactinol synthase 1 (*GolS1*)^41^. In addition to *HsfA2* and many *HSPs*, homologues of both *APX2* and *GolS1* genes were up regulated in the “low scald” fruit and could participate in the osmoprotection and ROS scavenging systems of the cells and prevent scald in response to cold stress (Table 3). Indeed, being involved in the response to a wide range of abiotic stresses, APX2 is a key enzyme of the redox homeostasis participating in the glutathione-ascorbate ROS scavenging cycle^56^, while GolS1 is involved in the synthesis of raffinose family oligosaccharides. In Arabidopsis galactinol and raffinose compounds can scavenge hydroxyl radicals to protect plant cells from oxidative damage caused by chilling^57^.

Protection against cold stress for “low scald fruit” was also probably enhanced by the expression of other genes involved in the maintenance of the cellular redox homeostasis. Apple genes coding for thioredoxin, glutaredoxin (GRX), glutathione-S-transferase (GST) and 2-oxoglutarate (2OG) or Fe(II)-dependent oxygenase were also relatively more expressed at harvest in “low scald” fruit. The Arabidopsis GRX gene *AtGRXS17* was shown to confer chilling stress tolerance in tomato fruit by increasing antioxidant enzyme activities and reducing H_2_O_2_ accumulation^58^. GSTs are directly involved in reducing oxidative damage as well as in resistance and adaptation to various abiotic stresses in plants^59^. In the same way, over expression of bZIP gene *HY5* and MYB transcription factors in “low scald” fruit could also participate in the maintenance of the cellular redox homeostasis through their role in anti-oxidant components synthesis and therefore promote cold acclimation of plants^18,42,43^.

The pre-storage acclimation treatment was sufficient in temperature and duration to reduce superficial scald, while preserving the fruit qualities (colour and firmness). A similar result was obtained by Moggia *et al*.^17^ with a step-wise cooling experiment. The acclimation treatment also delayed the repression of several *HSP* genes involved in abiotic stress response (Fig. 4c). This result indicates that *HSP* genes induction in 2014 was not specifically due to the progressive decrease in temperature three days before harvest but more probably to the previous warm period or by the combination of warm and cold periods (Fig. 3). This result supposes also that the increase in temperature in 2017 just before harvest was not long or intense enough to induce and maintain *HSP* gene expression until the end of the cold acclimation. Therefore, it could be interesting to test the post-harvest effect of a heat shock or a warm period followed by a cold acclimation on *HSP* gene expression and superficial scald development.

The expression profiles of *FAR* and *PPO*, coding respectively an α-farnesene synthase and a polyphenol oxidase often proposed to be involved in scald symptoms development, through the early phase of cold storage could not explain the efficiency of the acclimation treatment. In contrast, stress responsive genes expression profiles indicated differential fruit physiological status (Fig. 4c). PAL is the first enzyme of the polyphenol biosynthesis pathways leading to several groups of secondary metabolites such as lignins and flavonoids, including anthocyanins^60^. PAL regulation and ROS scavenging properties of the polyphenols led to the hypothesis that PAL was a key element in plant thermal stress acclimation^61^. PAL expression and activity has already been associated with chilling tolerance in several fruit species^62,63^. In particular, *PAL* induction of expression was observed in peel of citrus under cold stress and was attributed to a rapid adaptive response of the tissue to low temperature^64^. The rapid induction of *PAL* expression under classic cold or acclimation conditions is consistent with the observed increase in PAL enzyme when apple peel disc were chilled^65^. Probably because of milder temperatures, the acclimation treatment led to a lower induction of *PAL* and suggests a lower stress status for these fruit when compared with those subjected to classic cold conditions.

In addition to being a transcriptional biotic and abiotic stresses responsive marker, PR5 was shown to regulate physiological processes and interact with the ROS processing system^66^. PR5-osmotin helps in the accumulation of proline, an osmolyte which quenches reactive oxygen species and free radicals^67^. The overexpression of PR5-osmotin in chili pepper was shown to increase the activity of ascorbate peroxidase (APX) and superoxide dismutase (SOD) to detoxify the accumulated ROS^68^. Contrary to PAL, PR5 gene expression was not increased immediately but only after one week of cold storage (Supplementary Fig. S4). No consistent effect of acclimation on PR5 expression was observed at this stage. In potato under cold acclimation, the induction of osmotin-like proteins such as PR5 was progressive and increased 2 weeks after the treatment^69^. In this context, PR5 would be a marker of stress accumulation over the cold storage period. The benefit of acclimation on fruit stress status and *PR5* differential expression should be observed over a longer time frame.

It is now well established that ROS are essential messengers involved in redox signalling to regulate a wide range of processes, including responses to abiotic stress^70^. Thus cold acclimation, with milder temperatures when compared with classic cold conditions, could have been sufficient to affect cellular redox homeostasis and stimulate the ROS processing system^71^. The generated signal may have entrained acclimation and improved apple stress tolerance by physiological adaptations (Fig. 5). The benefit of the acclimation in reduced scald symptoms would have been persistent until the end of the long cold storage period.

This hypothesis supports the idea that the physiological status of fruit and in particular the status of its ROS processing system before cold storage could be very critical for further superficial scald development during shelf-life. It would also explain the fruit maturity effect on scald incidence observed with H2 and H3 harvest irrespective of the year. Indeed, some studies showed that delayed harvests increased the accumulation of anti-oxidant metabolites such as α-tocopherol, carotenoids, anthocyanin in apple peel, and reduced superficial scald^72^. Levels of antioxidant products such as glutathione and ascorbate, and related enzyme activities, superoxide dismutase (SOD), catalase (CAT), ascorbate peroxidase (APX) and glutathione peroxidase (GPX), were also found to increase during the ripening process of pear, peach and tomato^73–75^. This hypothesis is also in agreement with the theory that stress cross-tolerance is mediated through the ROS processing system and the associated regulation of gene expression through the redox signalling hub^76^ (Fig. 5). Therefore, it would be interesting to study the ROS processing system and its related gene network in response to pre-storage acclimation treatments and along the long cold storage of fruit.

## Conclusion and Perspectives

*In vivo* acclimation by pre-harvest thermal variations, including a warm period of several days, as well as post-harvest acclimation by a mild-cold period significantly reduced superficial scald development. Post-harvest thermotherapy treatments, which are short intense heat shock, are currently under evaluation in order to reduce post-harvest fungal infections. The success of this strategy supposes a non-antagonistic effect on other post-harvest disorders such as superficial scald. The correlation between the induction of HSF and HSP gene expression and the limitation of scald incidence suggests that thermotherapy treatments could limit scald. A post-harvest procedure combining both thermotherapy and cold acclimation could be even more efficient in preventing scald disorders.

This study revealed that HSP gene expression could be a potential marker to predict as early as harvest the risk of scald development during subsequent cold storage. Additional stress responsive genes identified at harvest (Table 3) or during the early days of cold storage, as well as orchard temperatures would increase the reliability of such a prediction which could support decisions for post-harvest treatments and/or cold storage management.

## Supplementary information

Supplementary Information.

Supplementary File S1.

Supplementary File S2.

Supplementary File S3.

Supplementary File S4.

## Data Availability

The microarrays data are available from the Gene Expression Omnibus database (https://www.ncbi.nlm.nih.gov/geo/) under the accession number GSE135863.
