# Supplementary material for: Pre-harvest climate and post-harvest acclimation to cold prevent from superficial scald development in Granny Smith apples
Source: Sci Rep. 2020 Apr 10;10:6180. doi: 10.1038/s41598-020-63018-3 (PMC7148358; doi:10.1038/s41598-020-63018-3)
Supplement: Supplementary file 1 — Supplementary Information. [file 41598_2020_63018_MOESM1_ESM.pdf]

Pre-harvest climate and post-harvest acclimation to cold prevent from superficial scald development in Granny Smith apples.

Mathieu Marc[1], Maryline Cournol[1], Sylvain Hanteville[1], Anne-Sophie Poisson[1], Marie-Charlotte Guillou[1], Sandra Pelletier[1], François Laurens[1], Christine Tessier[2], Claude Coureau[2,3], Jean-Pierre Renou[1], Mickaël Delaire[1] & Mathilde Orsel[1*]

[1]IRHS, Université d'Angers, AgroCampus Ouest, INRAE, SFR 4207 QuaSaV, 42 rue Georges Morel, 49071, Beaucouzé cedex, France

[2]Station d'expérimentation fruitière La Morinière, 37800 Saint-Epain, France

[3]Centre technique interprofessionnel des fruits et légumes (CTIFL), France

*Corresponding author: mathilde.orsel-baldwin@inrae.fr

Supplementary Fig. S1 Effect of year and fruit maturity on superficial scald severity

Supplementary Fig. S2 Additional pre-harvest climatic variables

Supplementary Fig. S3 Effect of acclimation on superficial scald severity

Supplementary Fig. S4 Effect of cold storage on relative gene expression (RT-qPCR)

Supplementary Table S1 Harvest dates

Supplementary Table S2 Primers used for RT-qPCR

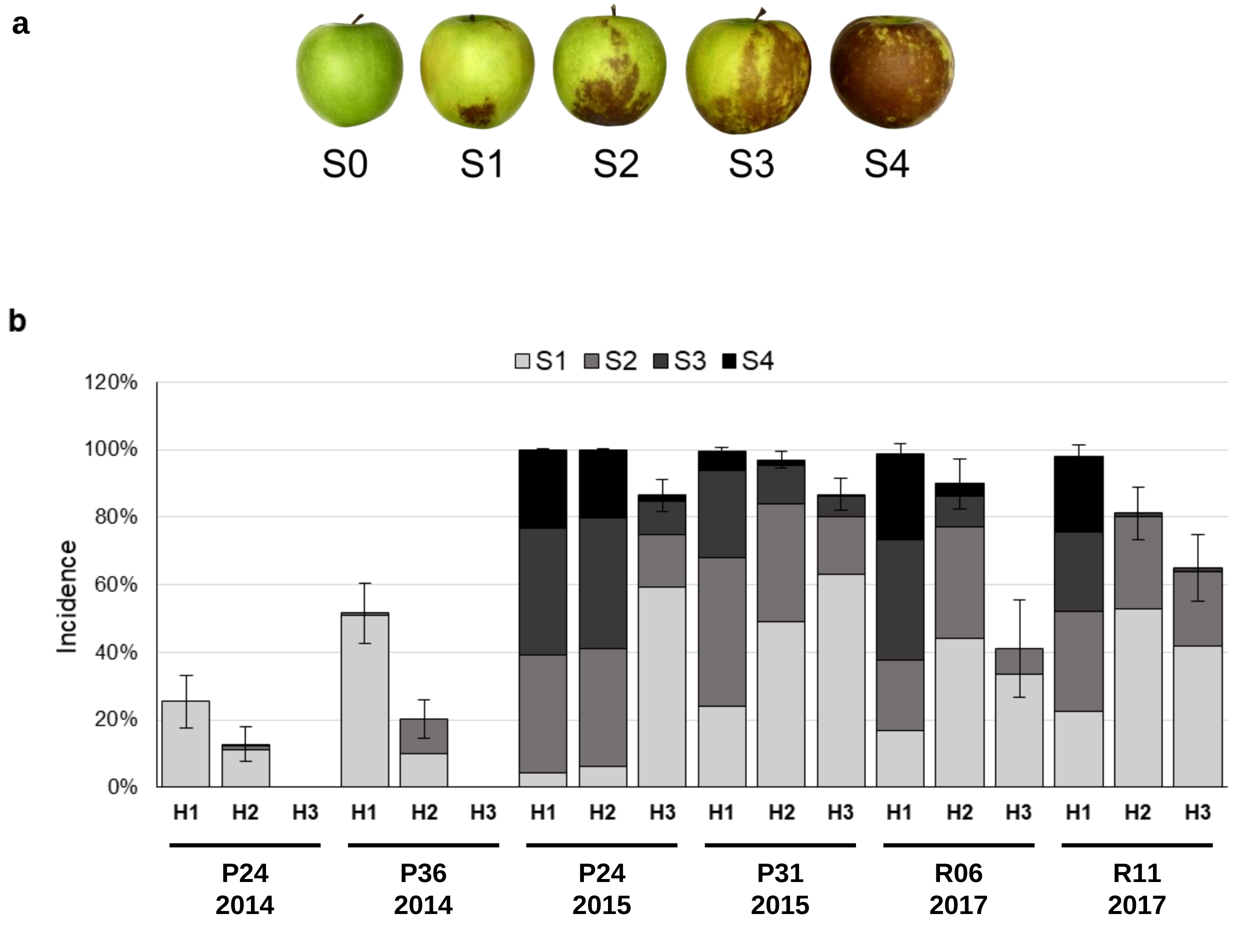

Supplementary Fig. S1 Effect of year and fruit maturity on superficial scald severity

(a) Superficial scald severity scale according to the relative surface area affected by symptoms as following: S0, no symptom; S1, > 0 % to 25 %; S2, > 25 % to 50 %; S3, > 50 % to 75 %; S4, > 75 % of affected surface area.

(b) Annual scald incidence and severity according to maturity at harvest. Fruit were harvested at three different maturity stages, early (H1), optimal (H2) or late (H3), from two different orchards each year in 2014, 2015 and 2017, and stored under cold controlled atmosphere for 5 to 6 months (see Fig. 1). Values are proportion of fruit per class, and binomial confidence interval for incidence (n = 100 to 200, α = 0.05).

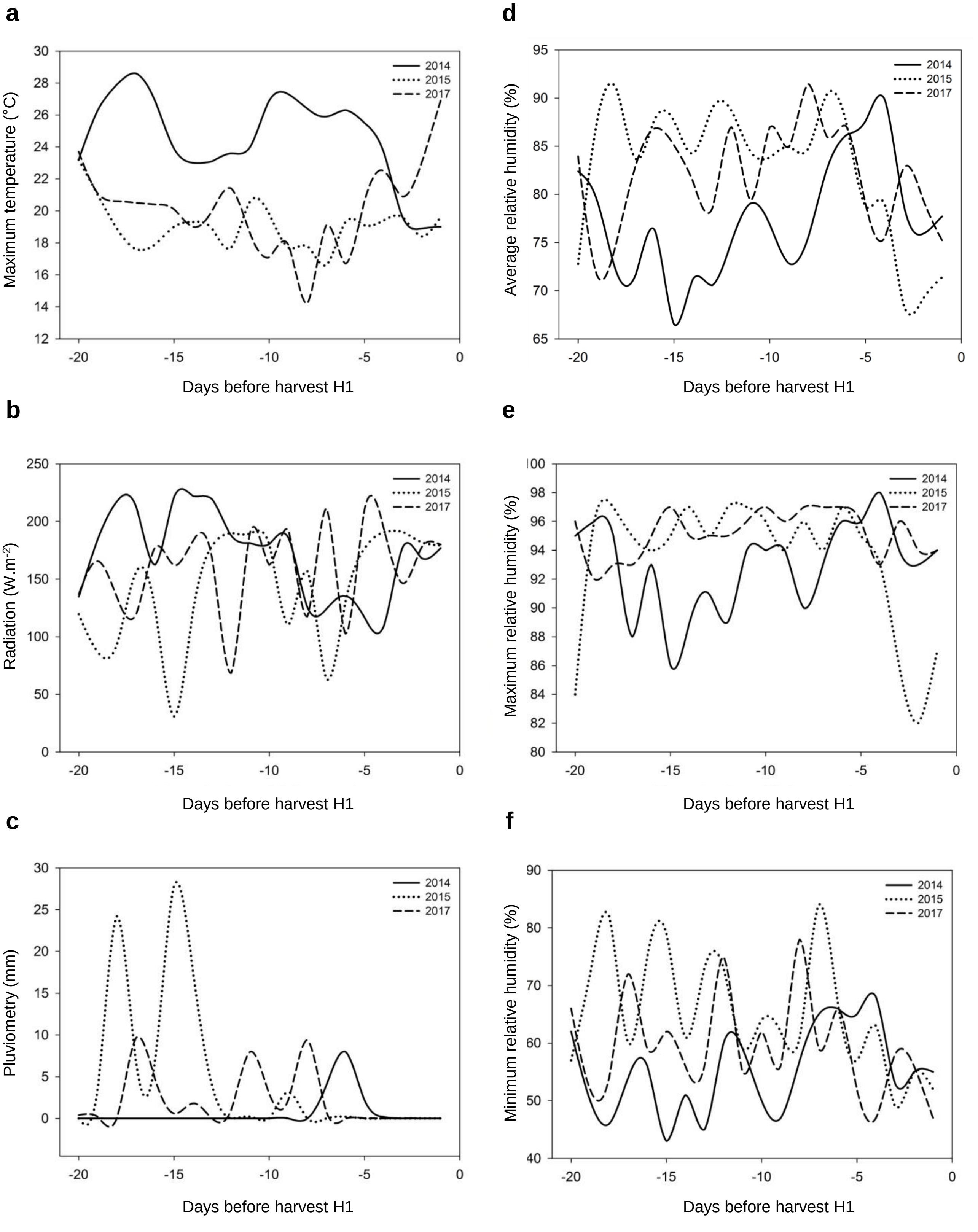

Supplementary Fig. S2 Additional pre-harvest climatic variables

Orchard climatic data were retrieved on a daily base, each year, for 20 d before early harvest H1. Mean and minimum daily temperatures (°C) are presented Fig. 2c and 2d; (**a**) maximum daily temperatures (°C), (**b**) Radiation (W m^{-2}), **(c)** Pluviometry (mm), (**d**) average, (**e**) maximum and (**f**) minimum relative humidity (%).

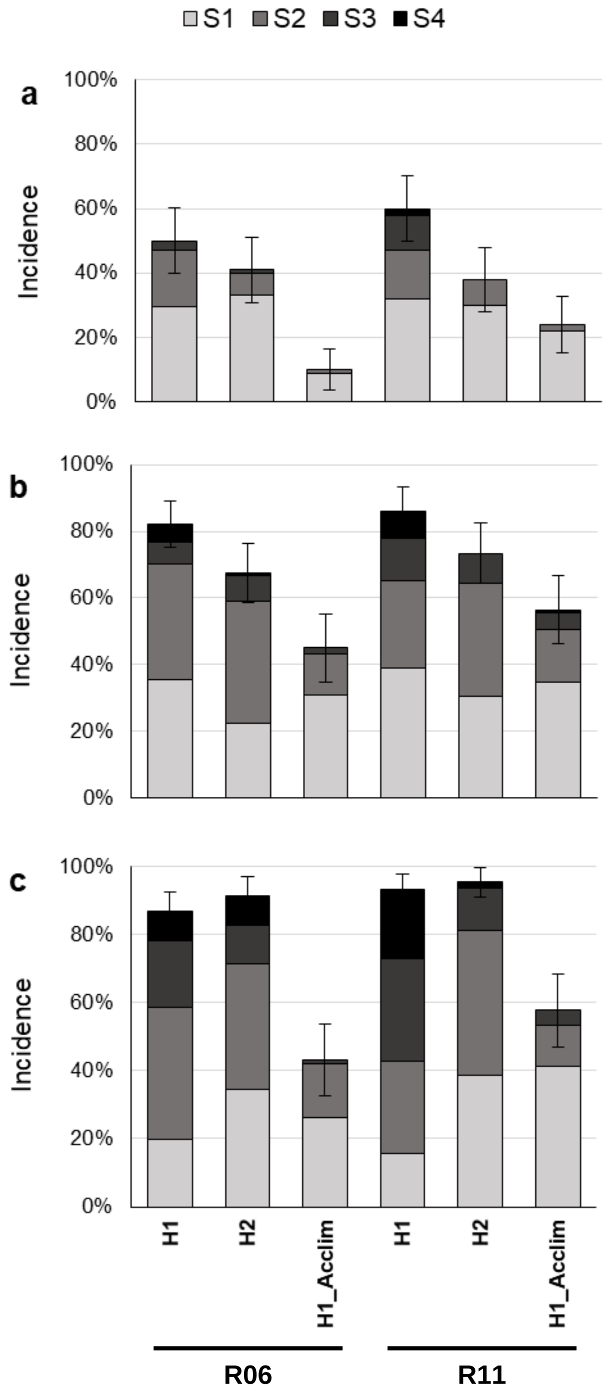

Supplementary Fig. S3 Effect of acclimation on superficial scald severity

Scald incidence and severity according to acclimation treatment (H1-acclim) or harvest stage (H1 and H2) (see Fig. 4a). Fruit were harvested from two different plots and stored under classic cold conditions for (**a**) 3 months, (**b**) 4 months and (**c**) 5 months. Severity scale according to the relative surface area affected by symptoms as following: S1, > 0 % to 25 %; S2, > 25 % to 50 %; S3, > 50 % to 75 %; S4, > 75 % of affected surface area. Values are proportion of fruit per class, and binomial confidence interval for incidence (n = 100, $\alpha = 0.05$).

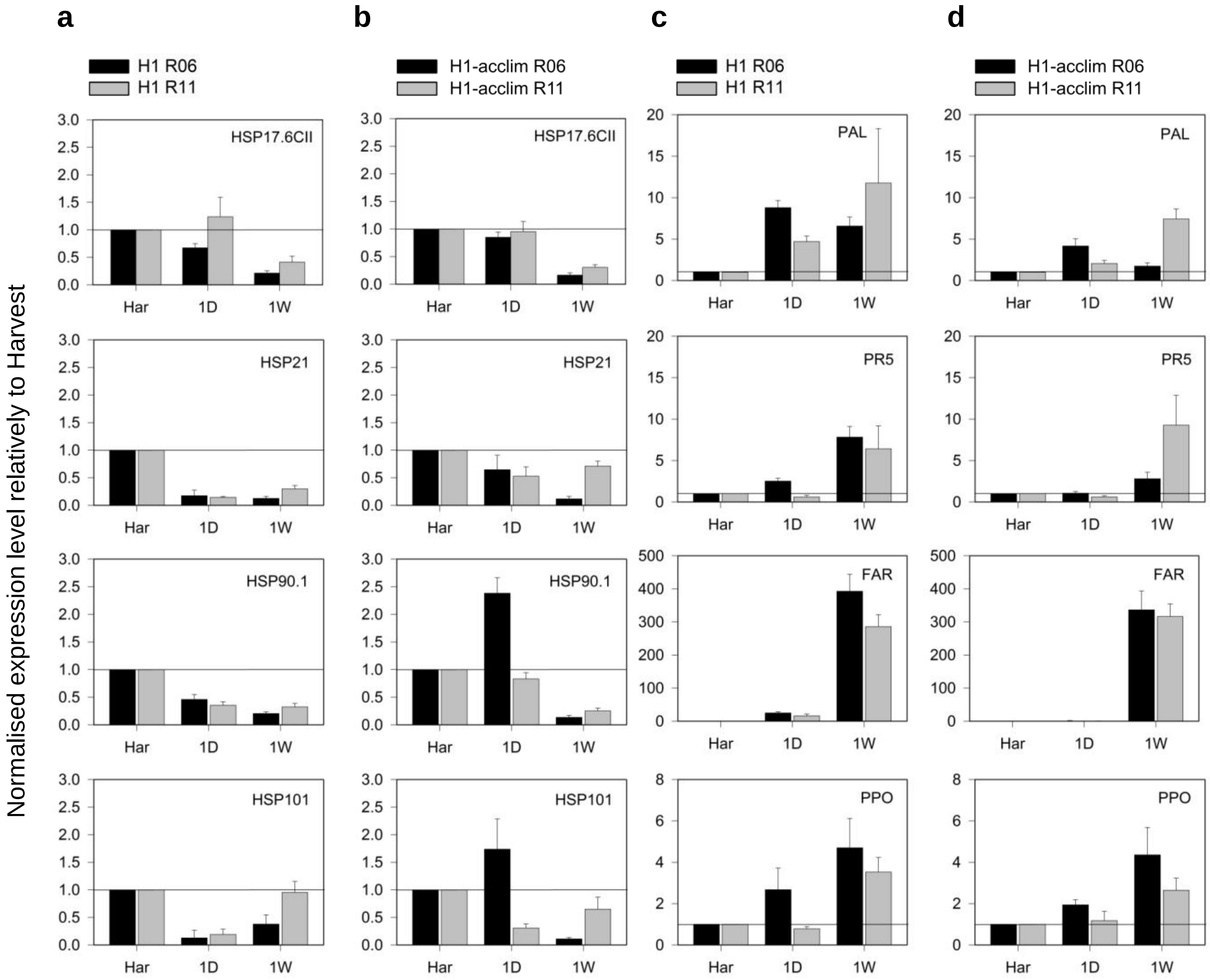

Supplementary Fig. S4 Effect of cold storage on relative gene expression (RT-qPCR)

Normalised relative gene expression in fruit peel samples after one day (1D) and one week (1W) of classic cold storage (H1, **a** and **c**) or acclimation (H1-acclim, **b** and **d**). Expression relatively to harvest stage (Har) for HSP17.6CII (*MD15G1053800*), HSP21 (*MD13G1108500*), HSP90.1 (*MD01G1208700*), HSP101 (*MD06G1201600*) (**a** and **b**), PAL (*MD12G1116700*), PR5 (MD04G1064200), FAR (*MD10G1311000*) and PPO (*MD10G1299400*) (**c** and **d**). Data are mean values ± SD of n = 3 for fruit collected in both orchards R06 and R11.

Supplementary Table S1 Harvest dates

	Starch index (SI)	Harvest dates 2014	2015	2017
**H1 (early)**	3	2014-09-25	2015-10-01	2017-09-25
**H2 (optimal)**	5	2014-10-06	2015-10-08	2017-10-03
**H3 (late)**	7	2014-10-20	2015-10-15	2017-10-10

Supplementary Table S2 Primers used for RT-qPCR

Type of gene	Short Name	MD gene	GDDH13 Annotation		Primer sequence
GOI	HSP17.6CII	MD15G1053800	Heat shock protein 17.6 kDa class II	F<br>R	GATGCGATTTCTGCTGTTTG<br>TCCCCGCATGATTACTCTCA
GOI	HSP21	MD13G1108500	Heat shock protein 21	F<br>R	CCAAGAGCTATGGGAGGTAC<br>TACCGGAAAGTCATGGAAGC
GOI	HSP40	MD10G1289200	DNAJ heat shock family protein	F<br>R	CCCAAAGACCCCTCCAAGAA<br>TTCACATAGCACATCGGCTC
GOI	HSP90.1	MD01G1208700	Heat shock protein 90.1	F<br>R	AATTCACCGGATGTTGAAGC<br>TGACATCCCAATTGACTCCA
GOI	HSP101	MD06G1201600	Heat shock protein 101	F<br>R	GCAGAAGTCGGATGTGTTGA<br>CCGGAACTGTTCTGCATTTT
GOI	PAL	MD12G1116700	PHE ammonia lyase 1	F<br>R	GGATCTCCTCAAAGTTGTCG<br>CTCACTCTCGCCATTTGTC
GOI	PR5	MD04G1064200	Thaumatin (PR-5)	F<br>R	AAAGGGGCTCGCATTTGG<br>CTTGGCATTGGAGGACACC
GOI	FAR	MD10G1311000	Terpene Synthase (TPS)	F<br>R	GATATTTTAGATGAGGCGAAAGC<br>GCGTTGATTTGCCATTTGAC
GOI	PPO	MD10G1299400	Polyphenol oxidase, chloroplastic	F<br>R	TGCCCGCCGCCTTCCAC<br>GCTCCATCGCTTTGTAGTATTTGTC
GOI	LOX	MD11G1023100	Lipoxygenase 1	F<br>R	GTTGCGTATGGGAAGGAATGG<br>GGTAGTAGTGGTTTACATAGTCAGTG
GOI	EXGT	MD16G1145200	Xyloglucan endotransglucosylase/hydrolase	F<br>R	GACGGCGTAGGAGTGGTAAG<br>AAACAACGAACACGACGAGA
GOI	IMS1	MD05G1155100	2-isopropylmalate synthase	F<br>R	GCCATGATCTGGACGAAAAG<br>GGCCACCTGATAAACCAATG
Ref	VDAC2	MD02G1185100	Voltage dependent anion channel 2	F<br>R	CGCCCAAAATTATTGAAGCA<br>ATCCCCAGCCATTGATTTTT
Ref	GAPDH	MD16G1111100	Glyceraldehyde-3-phosphate dehydrogenase C2	F<br>R	ATCAATGAAGGACTGGAGAGG<br>GTCAGGTCAACAACGGAAAC
Ref	EIF3K	MD15G1223700	Eukaryotic translation initiation factor 3K	F<br>R	GCAAGCAATCCAAGCCTATG<br>ACTTCGAGGTAGGACAATG
